# Assessment of Longitudinal Reproducibility of Mice LV Function Parameters at 11.7 T Derived from Self-Gated CINE MRI

**DOI:** 10.1155/2017/8392952

**Published:** 2017-02-22

**Authors:** Zhi Zuo, Anne Subgang, Alireza Abaei, Wolfgang Rottbauer, Detlef Stiller, Genshan Ma, Volker Rasche

**Affiliations:** ^1^Department of Internal Medicine II, University Hospital Ulm, Ulm, Germany; ^2^Department of Cardiology, Zhongda Hospital, Medical School of Southeast University, Nanjing, China; ^3^Core Facility Small Animal MRI, Medical Faculty, Ulm University, Ulm, Germany; ^4^In-Vivo Imaging, Drug Development, Boehringer Ingelheim Pharma Gmbh & Co. KG, Biberach, Germany

## Abstract

The objective of this work was the assessment of the reproducibility of self-gated cardiac MRI in mice at ultra-high-field strength. A group of adult mice (*n* = 5) was followed over 360 days with a standardized MR protocol including reproducible animal position and standardized planning of the scan planes. From the resulting CINE MRI data, global left ventricular (LV) function parameters including end-diastolic volume (EDV), end-systolic volume (ESV), stroke volume (SV), ejection fraction (EF), and left ventricular mass (LVM) were quantified. The reproducibility of the self-gated technique as well as the intragroup variability and longitudinal changes of the investigated parameters was assessed. Self-gated cardiac MRI proved excellent reproducibility of the global LV function parameters, which was in the order of the intragroup variability. Longitudinal assessment did not reveal any significant variations for EDV, ESV, SV, and EF but an expected increase of the LVM with increasing age. In summary, self-gated MRI in combination with a standardized protocol for animal positioning and scan plane planning ensures reproducible assessment of global LV function parameters.

## 1. Background

Recent developments in genetic engineering and surgical and pharmacological methods have led to a wide selection of small rodent animal models [1], allowing a better understanding of the underlying mechanisms of cardiovascular diseases and to study new therapeutic interventions. The diagnosis and management of cardiovascular diseases require accurate assessment of cardiac function [2]. The small size of the mouse heart (approximately 1/2000th the mass of a human heart), high heart rates (about 250–650 beats per minute [bpm], depending on the depth of anesthesia), and high respiratory rates (about 60–160 cycles per minute [cpm]) impose substantial challenges on phenotyping methods.

Echocardiography using high-frequency transducers has proven to be useful in estimating LV function in mice [3]. It is a quick and affordable technique. However, it intrinsically has a relatively low reproducibility since imaging conventionally is 1D (M-mode) or 2D and so relies on geometric assumptions for volume calculation. Further echocardiography as such is highly dependent on the experience of the user and thus showing high interexaminer variability. Furthermore, shadowing by the sternum and limits on spatial resolution prevent accurate analysis of the ventricles.

Magnetic resonance imaging (MRI) is a noninvasive technique that uses intrinsic tissue contrast and is capable of obtaining volumetric data of the heart and the vascular system. In numerous previous studies, cardiovascular magnetic resonance (CMR) imaging has proven to be an accurate technique and is considered the gold standard for noninvasive assessment of left ventricle (LV) function parameters in humans [4, 5]. The advantages of CMR are derived from the high soft tissue contrast between blood and myocardium, high spatial and temporal resolution, and the ability to acquire arbitrary slice orientations. On dedicated small animal systems, CMR is able to meet the requirements of the small-sized, fast-beating mouse heart and has thus emerged as the most accurate imaging modality currently available for deriving detailed, noninvasive assessments of cardiac structure and function in mice [6, 7]. With the recent developments of self-gated cardiac imaging [8], data acquisition has been dramatically simplified and cardiac imaging in small rodents is ready to become mainstay for assessment of cardiac function.

Where developmental studies have been done by Wiesmann et al. for assessing the cardiac function and mass between neonatal (day 3) and juvenile (week 16) age after birth [7], reproducibility studies especially of the self-gated techniques are limited. Recent studies include longitudinal assessment of cardiac function and mass at a limited number of time points [9, 10], application to rats [11–14], or low field-strength applications [15–18]. Further studies include assessment of the intra- and interobserver reproducibility [9], with Vanhoutte et al. applying the simplified self-gated imaging technique. Even though the investigations showed excellent reproducibility of cardiac function and left ventricular mass with rather low inter- and intraobserver variability, studies of the longitudinal reproducibility, which are important for assessment of the sensitivity of CMR for changes of cardiac parameters over time, have not been published, yet.

For assessment of the longitudinal reproducibility of CMR-derived cardiac parameters at 11.7 T, in this study a group of adult mice were followed for 360 days (postnatal day (PND) 91–PND 451) with a standardized imaging protocol for assessment of changes of cardiac parameters over time and the reproducibility of the imaging protocol.

## 2. Methods

### 2.1. Ethics Approval

Animal experiments were approved by the regional board of Tübingen and conducted according to German law for the welfare of animals and regulations for care and use of laboratory animals. All institutional and national guidelines for the care and use of laboratory animals were followed and approved by the appropriate institutional committees.

### 2.2. Animals

Female wild type mice (C57/B6, *n* = 5) were included in this study. Before the start of the study, the animals were acclimatized in a temperature-controlled environment for one week. Facility rooms were maintained at constant temperature (23°C), humidity (50% relative humidity), and 12 h light-dark illumination cycle. Access to food and tap water was ad libitum.

### 2.3. CMR Image Acquisition

#### 2.3.1. MRI Protocol

**Figure 1:**
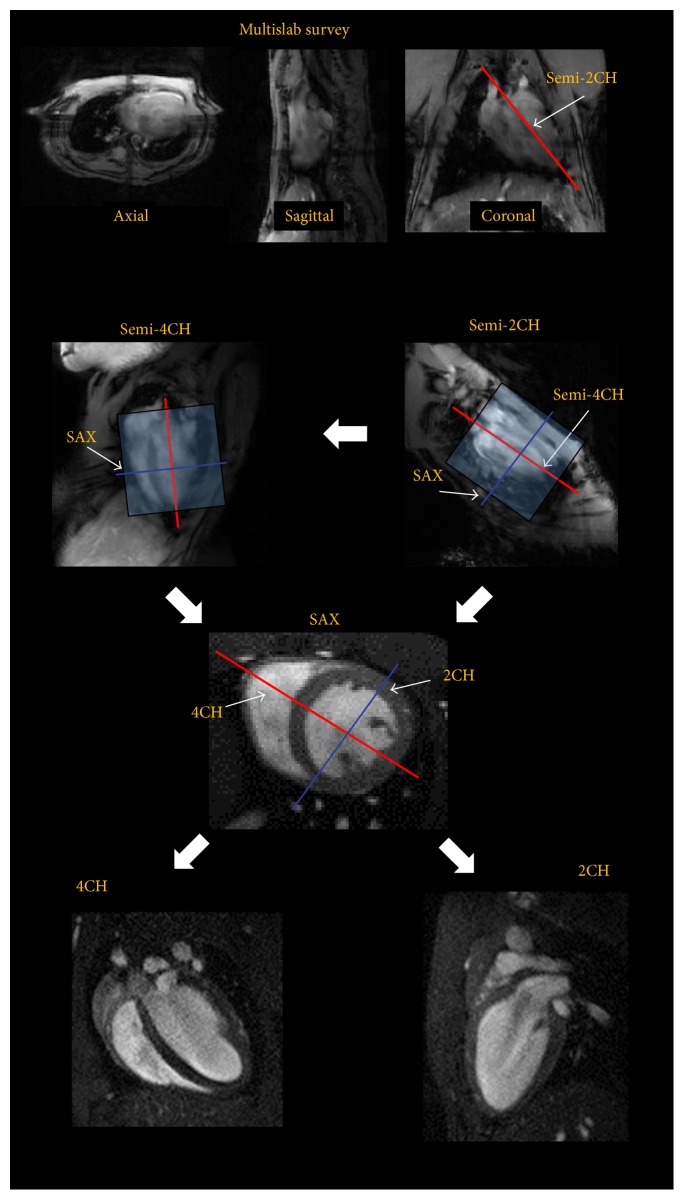
Imaging protocol for highly reproducible imaging of cardiac function and anatomy (2/4CH: 2/4-chamber, SAX: short axes orientations).

**Figure 2:**
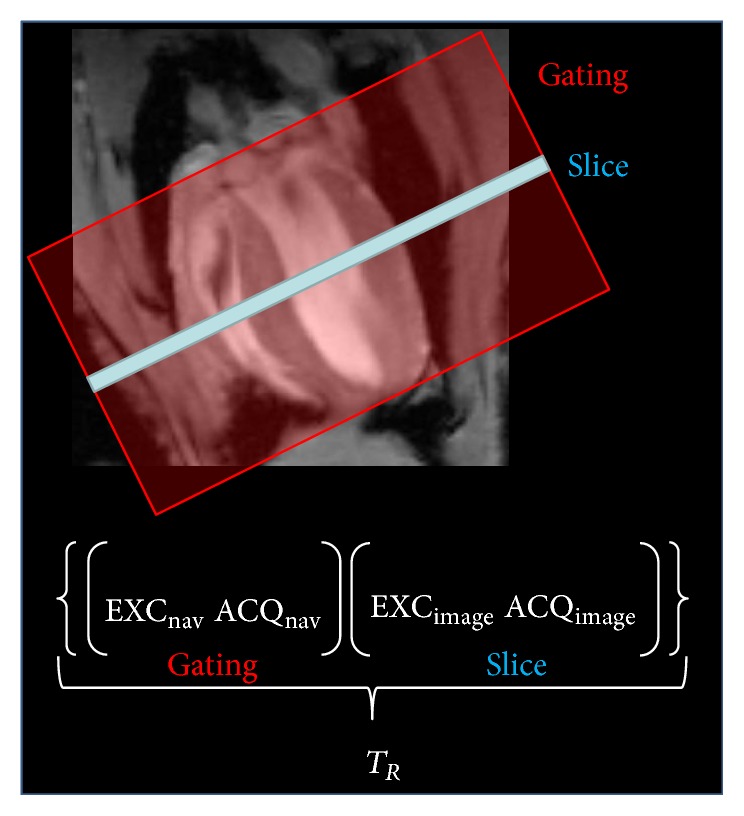
Principle of the IntraGate CINE imaging technique.

Imaging was performed on an 11.7 Tesla small animal MRI system (Bruker BioSpec 117/16, Germany). Data were obtained with a dedicated four-element array-coil optimized for imaging the mouse heart (Bruker BioSpec, Ettlingen, Germany). The MR imaging protocol (Figure 1) comprised a multislab survey acquisition in axial, coronal, and sagittal orientation, followed by two long-axis CINE scans in semi-two-chamber (semi-2CH) and semi-four-chamber (semi-4CH) geometry. The semi-2/4CH images were used to plan the subsequent stack of short axes orientations (SAX). The number of short axis slices was adopted to ensure full coverage of the ventricles in end-diastole. Final 2- and 4-chamber orientations were planned on the SAX data. All cine acquisitions were performed using a self-gating approach (IntraGate©, Bruker Biospin, Ettlingen, Germany). The principle of the self-gating technique is depicted in Figure 2. In short, the self-gating technique is a modified retrospectively gated Fast Low Angle Shot (FLASH) [19] imaging approach. The major modification results from splitting up the acquisition of each *k*-space line into two subsequently performed acquisitions. During the first acquisition a navigator signal without phase encoding is generated, and with the subsequent acquisition a single line in *k*-space is encoded as well known from the conventional FLASH technique. The navigator signal is derived from the magnitude and phase of few initial points of the free induction decay (FID) after low flip-angle excitation. To maximize heart-phase related navigator signal alterations, a volume covering almost the complete ventricles is chosen for generation of the navigator signal. To ensure sufficient data for the subsequent multiphase reconstruction, the acquisition of the complete data set is repeated several times (number of repetitions). During retrospective reconstruction, the acquired data is sorted according to the identified cardiac and respiratory phase thus enabling the reconstruction of cardiac- and respiratory-phase resolved CINE data. Excellent myocardium-blood contrast is obtained by sequential acquisition of the slices thus minimizing the repetition time (TR) and hence saturation effects of blood. Detailed parameters of the different sequences are summarized in Table 1.

#### 2.3.2. Imaging Protocol

All animals were scanned under isoflurane anesthesia. For initiation of the anesthesia, 5% isoflurane concentration in room air was used. For maintaining the anesthesia during the MRI, the isoflurane concentration was adapted to a value resulting in a respiratory frequency between 50 and 70 respiratory cycles per minute. Depending on the animal, isoflurane concentrations between 1% and 1.5% were required. During scanning the respiratory rate was monitored and the temperature of the animals was controlled by water heating of the animal bed. The animals were positioned in prone position carefully taking care for straight alignment of the animal with the animal support. The respiratory cushion was placed between the abdominal wall and the animal cradle at the level of the liver region to minimize its impact on the positioning of the animal in the target area.

All animals were followed over 360 days and scanned following the MRI protocol mentioned above at 9 time points on 6 different days (PND 91, PND 93, PND 95, PND 151, PND 211, and PND 451 after birth). On PND 91, PND 93, and PND 95, the entire protocol was performed twice within 2 hours, yielding 9 measurements per animal in total. Between the subsequently performed scans on PND 91, PND 93, and PND 95, the animals were completely taken out of the scanner and repositioned.

### 2.4. CMR Image Analysis

All data were carefully analyzed regarding image quality. Data sets with significant artifacts caused by unexpected animal motion in at least a single slice were excluded. In this study no rescanning of corrupted slices was performed to ensure the same imaging protocol in all animals. LV global function parameters were evaluated by tracing epicardial and endocardial borders in end-diastole and end-systole. Contours were manually identified in all short axis images from base to apex. During delineation of the endocardial contour, papillary muscles were included as part of the myocardial wall. The apical slice was identified as the most distal segment that contains LV cavity and myocardium. The basal slice was defined as the most proximal segment located above the tips of the papillary muscles at the chordae tendineae. End-diastolic volume (EDV), end-systolic volume (ESV), and stroke volume (SV) were determined and LV ejection fraction (EF) and end-diastolic left ventricular mass (LVM) derived. The average time for image postprocessing was about 15 min per animal. All image analyses were done blinded using the freely available software Segment version 1.9.1 (http://segment.heiberg.se) [20].

### 2.5. Statistical Analysis

The data were used for assessing the (a) reproducibility or interexperiment variability of the proposed imaging approach, (b) the variability of the function parameters within a group, and (c) the longitudinal changes of the function parameters over one year.

Regarding (a) the assessment of the reproducibility of the suggested imaging protocol was performed based on the comparison of the function parameters of the subsequent acquired scans performed on PND 91, PND 93, and PND 95.

The mean and standard deviation were computed for each parameter and each measurement point. Statistical significance of differences between subsequent measurements was assessed applying a two-way ANOVA test with *p* values of *p* ≤ 0.05 being considered significant. The agreement between two measurements was assessed by mean of Bland-Altman analysis. Coefficient of variance (CoV) was calculated for assessing the variability within repeated measurements.

Regarding (b) intragroup variability of LV function parameters was assessed for all investigated parameters (EF, EDV, ESV, SV, and LVM) separately for all 9 time points, calculation of the respective CoV. The mean intragroup variability was derived for each parameter as the mean of the individual CoV of each of the 9 measurements.

Regarding (c) longitudinal variability was assessed over 360 days. ANOVA test was used to investigate the significance of changes over the 360d period for all derived LV parameters. For PND 91, PND 93, and PND 95, only the first measurement was included in the ANOVA analysis. CoV of each LV parameter was computed for each mouse.

The CoV was calculated as the fraction of standard deviation and mean of the respective function values. The reproducibility of the investigated parameters was classified according to the CoV value to excellent (CoV < 10%), very good (CoV ≥ 10% and <15%), good (CoV ≥ 15% and <20%), moderate (CoV ≥ 20% and <25%), acceptable (CoV ≥ 25% and <30%), and poor (CoV ≥ 30%).

ANOVA analysis was performed with SPSS 17.0 (IBM, USA). Bland-Altman test was performed with MedCalc 15.8 (MedCalc Software bvba, Belgium).

## 3. Results

The average total scan time resulted in about 30 minutes per animal. Mean respiratory and heart rates of the animals were 60 ± 8 (min = 47, max = 78) cpm and 398 ± 68 (min = 296, max = 565) bpm. All scans could be completed in all animals. In total, 7 out of the 45 image stacks were excluded from further analysis due to at least one motion corrupted slice in the short axes image stack.

**Figure 3:**
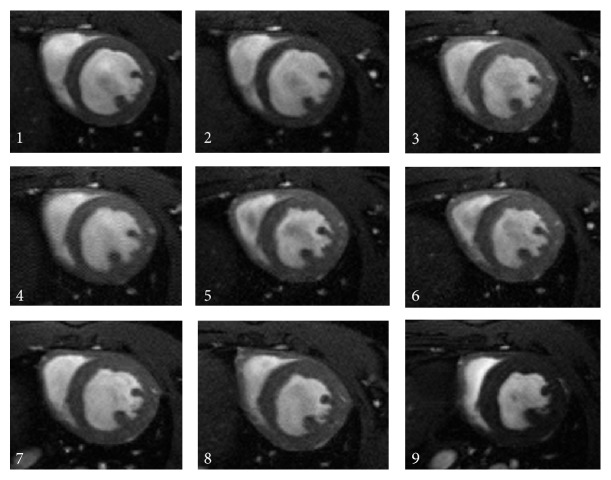
End-diastolic CINE magnetic resonance short axis images, acquired at a mid-ventricular level at measurement 1 of day 91 (1), measurement 2 of day 91 (2), measurement 1 of day 93 (3), measurement 2 of day 93 (4), measurement 1 of day 95 (5), and measurement 2 of day 95 (6), day 161 (7), day 211 (8), and day 451 (9) after birth.

Figure 3 shows short axis images acquired at mid-ventricular level along the long axis of the heart for all acquired time points exemplarily for one animal. Even though each of the scans was performed after completely repositioning of the animal and independent scan planning, the images indicate the high reproducibility of the investigated technique.

### 3.1. Reproducibility of the Imaging Protocol

**Figure 4:**
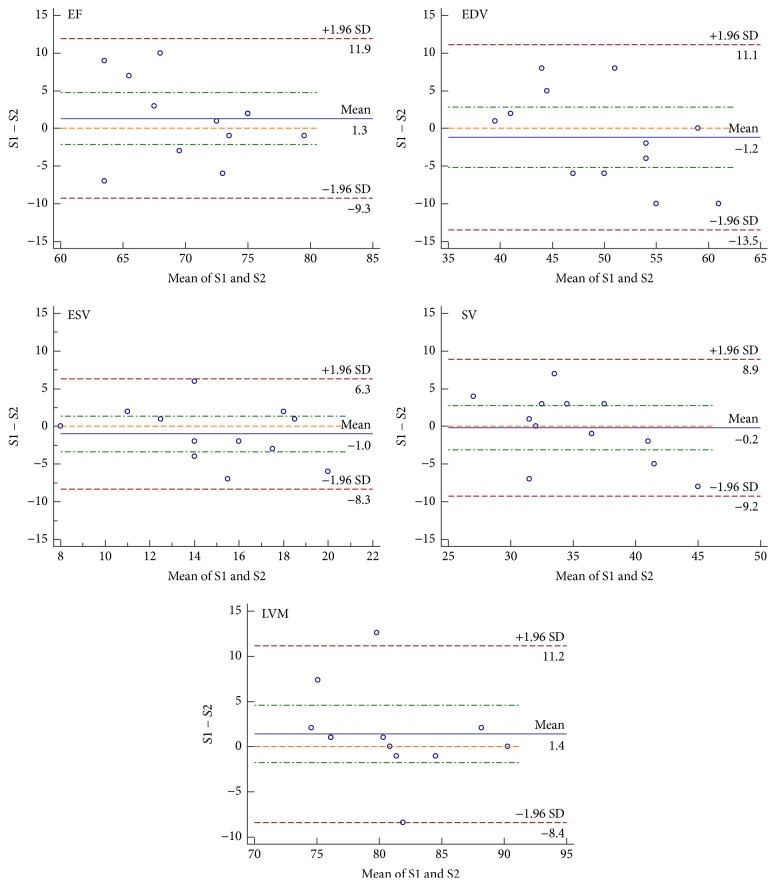
Bland-Altman plots of the differences between the subsequent measurements (S1, S2) acquired the same day.

For all investigated parameters, the mean ± SD, Bland-Altman analysis, *p* value, and CoV resulting from the comparison of the two subsequent measurements are summarized in Table 2. The respective Bland-Altman plots are provided in Figure 4. Between the two subsequent measurements acquired the same day, no significant differences were observed for all investigated parameters (dEF = 1.33%, dEDV = −1.17 ml, dESV = −1.00 ml, dSV = −0.17 ml, and dLVM = 1.40 mg). For EF and LVM excellent, for EDV and SV very good, and ESV moderate reproducibility was observed.

### 3.2. Intragroup Variability

The intragroup variability for all measurements and the respective mean values are provided in Table 3. Similar to the reproducibility measurements, intragroup variability was excellent for EF and LVW and very good for EDV and SV, but only moderate for the ESV.

### 3.3. Longitudinal Variability

**Figure 5:**
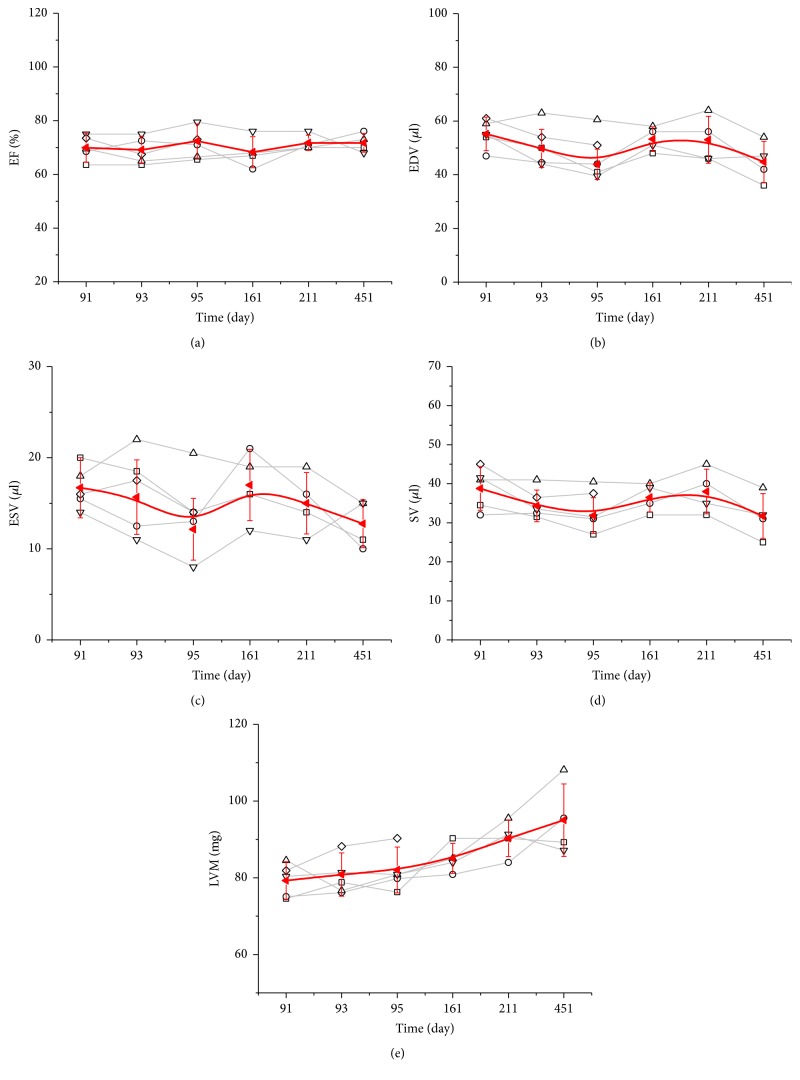
Individual (light) and mean (bold, red) left ventricular function parameters and mass for the six acquisition time points.

The longitudinal outcome for the investigated parameters is shown in Figure 5. From the longitudinal mean values, it becomes quite obvious that the EF is by far the most reliable parameter and EDV, ESV, and SV show more variation. For the LVM a longitudinal increase is observed.

The observations are confirmed by the ANOVA analysis presented in Table 4. Significant changes were observed in LVM only, showing increased values on PND 451. All other investigated parameters do not show significant variation over the one-year term.

CoV values over all time points for each investigated mouse are shown in Table 5. Longitudinal reproducibility was excellent for the EF, very good for EDV and SV, and moderate for the ESV.

## 4. Discussion

With the increasing use of small animal model in cardiovascular research, there is an increasing demand in noninvasive methods for assessing anatomy and function of the heart and to characterize myocardial tissue. For longitudinal studies, it is of utmost importance to ensure high reproducibility of the applied method to ensure sensitive identification of different outcomes even in rather small group sizes.

Currently, there is no established gold standard for assessment of cardiac function for small animals. Even though echocardiography is frequently applied, an only limited reproducibility for cardiac function parameters has been reported [21]. Factors such as usage of models for final calculation of volumetric data from few two-dimensional slices limit the accuracy and reproducibility of the technique. This is especially obvious in many animal models of cardiovascular disease showing abnormal ventricular geometry or heterogeneously regional cardiac function [22].

With CMR a versatile imaging tool is available, which enables noninvasive assessment of cardiac anatomy and function, and in combination with advanced imaging techniques has the potential to enable myocardial tissue characterization. In direct comparison with high-frequency echocardiography, MRI appears especially advantageous in animals cardiac pathologies impacting left ventricular geometry of regional function, cases in which echocardiography often lacks accuracy [22]. In particular, the full-volume acquisition possible with MRI was shown to yield improved reproducibility when compared with echocardiography [12]. There is only a small body of literature comparing MRI and echocardiography in the same animal model but there is strong evidence that the better delineation of the trabecular structures and in general the better contrast enable more accurate assessment of ventricular volumes and wall thickening [22, 23].

Cardiac MRI (CMR) in small animals was known as a cumbersome procedure suffering from low SNR, high respiratory and cardiac rates, distorted ECG signals by the magnetohydrodynamic effect, and currents introduced by the rapid gradient switching making the required cardiac synchronization difficult. With the advent of ultra-high-field MR systems SNR and hence spatial and temporal resolution of the CMR substantially increased over the last decade enabling high-quality assessment of cardiac function [24]. With the recently introduced and now widely available self-gating cardiac imaging techniques [8] CMR in small animals becomes more readily available and is increasingly applied for longitudinal studies of cardiac function. Some data had to be excluded due to motion of the animal during data acquisition. Even though not done during this study for keeping the protocol consistent, the acquisition of single corrupted slices can easily be repeated for obtaining sufficient image quality in all animals.

With the increasing use of CMR, knowledge of its reproducibility is gaining importance. Reproducibility studies of murine CMR have proved excellent intra- and interobserver variability, and correlation of CMR-derived left ventricular mass with postmortem measurements from ECG-synchronized acquisitions [24, 25]. Recently, Vanhoutte et al. reported high intra- and interobserver reproducibility and low interexperiment variability based on two subsequently performed measurements within 24 h with recent self-gating CMR protocols [9].

The goal of the presented study was to investigate the long-term reproducibility of cardiac function parameters derived from self-gated CMR. With the suggested imaging protocol, the interexperiment, intragroup, and longitudinal reproducibility could be quantified. For all measurement, scanning was performed according to a standardized protocol including animal positioning, scan orientation planning, and data evaluation. From the initial 6 measurements (PND 91/93/95, two measurements each day) excellent reproducibility was obtained for EF and LVM, very good for the EDV and SV, and moderate reproducibility for the ESV. In all cases no significant differences were found between the six measurements proving rather high interexperiment reproducibility. The observed values agree well with Vanhoutte et al. showing slightly lower variability for LVM, SV, and EF. Intragroup variability was assessed independently for each time point. With mean values between 7.38 (dEF) and 24.12 (dESV) the resulting intragroup variability was similar to the interexperiment variability. Long-term assessment of the variability (PND 91–PND 451) revealed highly reproducible cardiac function parameters over a one-year period and sensitive identification of even small changes as shown for the LVM, where a significant increase over time could be identified, while all function parameters remained unchanged.

From the data presented it is obvious that the EDV and the LVM can be quantified with highest fidelity and resulting interexperiment variability is excellent. ESV resulted as most critical parameter but still showed moderate (<25%) variability. This may be caused by several reasons. As the temporal resolution is limited and the cardiac cycle was only resolved with 20 cardiac phases, the rather high variability of the heart rate introduced different accuracy in defining the end-systolic phase. Furthermore, due to flow artifacts within the LV cavity and the smaller end-systolic volumes, errors introduced by partial volumes effect or erroneous contoured endocardial borders especially in the most apical and basal slices introduce a larger absolute error in the derived volumes as for the EDV.

The resulting low interexperiment and intergroup variability indicate that, by using a standardized animal positioning, scan orientation planning, and data analysis approach, excellent reproducibility can be achieved in murine CMR even for long-term studies.

## 5. Conclusions

In conclusion, considering the good inter- and intraobserver reproducibility reported by other groups [9] and the excellent long-term reproducibility shown in this study, murine CMR appears as an excellent tool for longitudinal monitoring of disease progression in cardiac diseases.

## Figures and Tables

**Table 1:** Acquisition parameters of the investigated MR protocol (MS survey: multistack survey; 2/4CH: two/four-chamber; SAX: short axes orientations; *T*_ACQ_: total acquisition time).

	MS survey	Semi-2CH	Semi-4CH	SAX	2CH	4CH
*T*_*E*_/*T*_*R*_ [ms]	1.25/23.5	1.25/20	1.25/20	1.0/5.75	1.0/5.75	1.0/5.75
Gated	No	Yes	Yes	Yes	Yes	Yes
Phases	NA	10	10	20	20	20
In-plane resolution [*µ*m]	117 × 117	117 × 117	117 × 117	117 × 117	117 × 117	117 × 117
Slice thickness [*µ*m]	1000	1000	1000	500	500	500
Number of repetitions	NA	75	75	200	200	200
*T*_ACQ_ [s]	47.185	40.4	40.4	1305.604	163.2	163.2

**Table 2:** Interexperiment comparison of the reproducibility and variability of cardiac function parameters and left ventricular mass (LVM).

	1st measurement	2nd measurement	Bias	95% CI	*p*	CoV
EF (%)	71.17 ± 4.99	69.83 ± 6.38	1.33 ± 5.42	−2.11~4.77	0.583	7.68
EDV (*µ*l)	49.42 ± 5.82	50.58 ± 9.03	−1.17 ± 6.28	−5.16~2.82	0.644	12.56
ESV (*µ*l)	14.42 ± 3.32	15.42 ± 4.38	−1.00 ± 3.72	−3.36~1.36	0.496	24.92
SV (*µ*l)	35.25 ± 4.29	35.42 ± 6.73	−0.17 ± 4.63	−5.16~2.82	0.935	13.10
LVM (mg)	81.46 ± 4.95	80.06 ± 6.19	1.40 ± 4.99	−1.77~4.57	0.554	6.18

**Table 3:** CoV (%) of left ventricular function parameters and left ventricular mass (LVM) for all investigated time points and the resulting mean value.

	CoV (EF)	CoV (EDV)	CoV (ESV)	CoV (SV)	CoV (LVM)
PND 91 1st measurement	4.90	11.01	20.55	9.70	4.03
PND 91 2nd measurement	10.02	10.27	15.18	18.29	8.37
PND 93 1st measurement	9.25	13.24	25.36	13.54	6.90
PND 93 2nd measurement	5.54	16.61	30.00	9.94	7.80
PND 95 1st measurement	6.33	14.81	28.91	13.28	7.18
PND 95 2nd measurement	13.01	10.38	30.99	17.96	9.06
PND 161	8.49	8.59	23.03	10.13	4.62
PND 211	4.00	16.45	22.44	15.04	5.29
PND 451	4.88	17.06	20.63	18.07	9.94
Mean	7.38 ± 2.99	13.16 ± 3.21	24.12 ± 5.19	13.99 ± 3.58	7.02 ± 2.03

**Table 4:** ANOVA analysis of longitudinal variability of LV parameters. Significant differences were observed only in the left ventricular mass.

	PND 91	PND 93	PND 95	PND 161	PND 211	PND 451	*p*
EF (%)	71.6 ± 3.51	68.6 ± 6.35	72.25 ± 4.57	68.25 ± 5.80	71.75 ± 2.87	71.75 ± 3.5	0.680
EDV (*µ*l)	52.4 ± 5.77	51.4 ± 6.80	45.25 ± 6.70	53.25 ± 4.57	53 ± 8.72	44.75 ± 7.63	0.274
ESV (*µ*l)	15 ± 3.08	16.4 ± 4.16	12.75 ± 3.69	21 ± 7.12	20.75 ± 3.77	18.75 ± 5.74	0.118
SV (*µ*l)	37.6 ± 3.65	35.2 ± 4.76	32.75 ± 4.35	36.5 ± 3.70	38 ± 5.72	31.75 ± 5.74	0.321
LVM (mg)	79.38 ± 3.20	81.9 ± 5.65	81.9 ± 5.88	85.05 ± 3.93	90.3 ± 4.77	95.03 ± 9.45	0.006

**Table 5:** CoV (%) of left ventricular function parameters for each investigated mouse over the 9 time points.

	CoV (EF)	CoV (EDV)	CoV (ESV)	CoV (SV)
Mouse 1	6.53	13.80	21.96	13.20
Mouse 2	7.09	12.29	24.72	9.70
Mouse 3	3.33	4.54	9.67	5.04
Mouse 4	4.55	14.29	23.74	13.17
Mouse 5	5.14	11.30	17.14	12.52
Mean	5.33 ± 1.51	11.25 ± 1.51	19.45 ± 6.19	10.73 ± 3.49

## Abbreviations

2CH: Two-chamber
4CH: Four-chamber
ANOVA: Analysis of variance
bpm: Beats per minute
cpm: Cycles per minute
CINE: As in cinema
CMR: Cardiovascular magnetic resonance
CoV: Coefficient of variance
ECG: Electrocardiography
EDV: End-diastolic volume
EF: Ejection fraction
ESV: End-systolic volume
FID: Free induction decay
FLASH: Fast Low Angle Shot
LV: Left ventricle
LVM: Left ventricle mass
MR: Magnetic resonance
MRI: Magnetic resonance imaging
MS: Multistack
PND: Postnatal day
SAX: Short axes orientations
*s*_*D*_: Slice thickness
SD: Standard deviation
SNR: Signal-to-noise ratio
SV: Stroke volume
*T*_ACQ_: Total acquisition time
TE: Echo time
TR: Repetition time.
